# Quantitative refractive index distribution of single cell by combining phase-shifting interferometry and AFM imaging

**DOI:** 10.1038/s41598-017-02797-8

**Published:** 2017-05-31

**Authors:** Qinnan Zhang, Liyun Zhong, Ping Tang, Yingjie Yuan, Shengde Liu, Jindong Tian, Xiaoxu Lu

**Affiliations:** 1 Nanophotonic Functional Materials and Devices, South China Normal University, Guangzhou, 510006 China; 2 College of Optoelectronic Engineering, Shenzhen University, Shenzhen, 518060 China

## Abstract

Cell refractive index, an intrinsic optical parameter, is closely correlated with the intracellular mass and concentration. By combining optical phase-shifting interferometry (PSI) and atomic force microscope (AFM) imaging, we constructed a label free, non-invasive and quantitative refractive index of single cell measurement system, in which the accurate phase map of single cell was retrieved with PSI technique and the cell morphology with nanoscale resolution was achieved with AFM imaging. Based on the proposed AFM/PSI system, we achieved quantitative refractive index distributions of single red blood cell and Jurkat cell, respectively. Further, the quantitative change of refractive index distribution during Daunorubicin (DNR)-induced Jurkat cell apoptosis was presented, and then the content changes of intracellular biochemical components were achieved. Importantly, these results were consistent with Raman spectral analysis, indicating that the proposed PSI/AFM based refractive index system is likely to become a useful tool for intracellular biochemical components analysis measurement, and this will facilitate its application for revealing cell structure and pathological state from a new perspective.

## Introduction

Cell refractive index, an intrinsic optical parameter, which is likely to help with both basic understanding of cell function and interpretation of pathological state, provides not only the intracellular mass and concentration, but also important insight for various biological models. Further, quantitative refractive index measurement of single cell, shows a great application potentiality in the research of cytobiology and disease diagnosis^1–4^. Early measurement techniques achieved only the average refractive index of a cell population suspended on the medium, in which the suspended cell was assumed to be homogeneous with the same refractive index, and the average cell refractive index can be determined by measuring either the refractive index change using interference refractometry or the optical density using optical densitometry^5–7^, so it was impossible to present the refractive index distribution information. After that, the effective refractive index model of single cell refractive index was proposed, in which a cell was treated as a container filled with a protein solution, the effective refractive index was defined as *n*_*ce*_ = *n*_0_ + *αC*, where *n*_0_ was the refractive index of water or dilute salt solution, *α* was the specific refraction increment and *C* was the mass density of protein in per deciliter, the main drawback of this technique was the assumption of a single living cell as a spherical object filled with a protein solution^4^. Further, several measurement techniques of cell refractive index distribution were developed, such as the quantitative phase imaging^8–10^ and tomographic phase microscopy (TPM)^9^. In the former, due to the decoupling procedure was needed with the aim of measuring separately the integral refractive index and the cellular thick, so it was assumed that the sample was only a spherical object. In the latter, due to the inhomogeneous refractive index distribution of biological cell, the 3-D mapping of refractive index in living cell can be achieved by performing the inverse calculation of scattering field, but the measurement accuracy of refractive index depended on the information amount of scattering field collection, and the spatial resolution at the subcellular level was only microscale in both the transverse and longitudinal directions. Recently, based on the transport of intensity equation (TIE)^11–13^, the refractive index distribution of single cell can be achieved, but the difference of refractive index between different wavelengths induced by the broadband illumination source cannot be ignored.

In this paper, we constructed a quantitative refractive index distribution of single cell measurement system, in which the cell quantitative phase and morphology are respectively achieved with high accuracy phase-shifting interferometry (PSI) technique^14, 15^ and atomic force microscope (AFM) imaging. As we know, due to several specific advantages, such as high accuracy, full-field, rapid speed, non-intervention, optical phase-shifting interferometry(PSI) technique has been widely utilized in phase retrieval of living cell^16–18^, in which the accurate phase map of single cell can be achieved with PSI technique. Moreover, it was reported that AFM imaging has several specific advantages in the morphology measurement of single cell, such as nanoscale resolution, non-intervention and simple sample preparation procedure^19–21^. In this study, combining PSI technique and AFM imaging, we intend to construct a PSI/AFM based refractive index measurement system and then achieve the quantitative refractive index distribution of single cell.

## Methods

### PSI/AFM based refractive index measurement system

**Figure 1:**
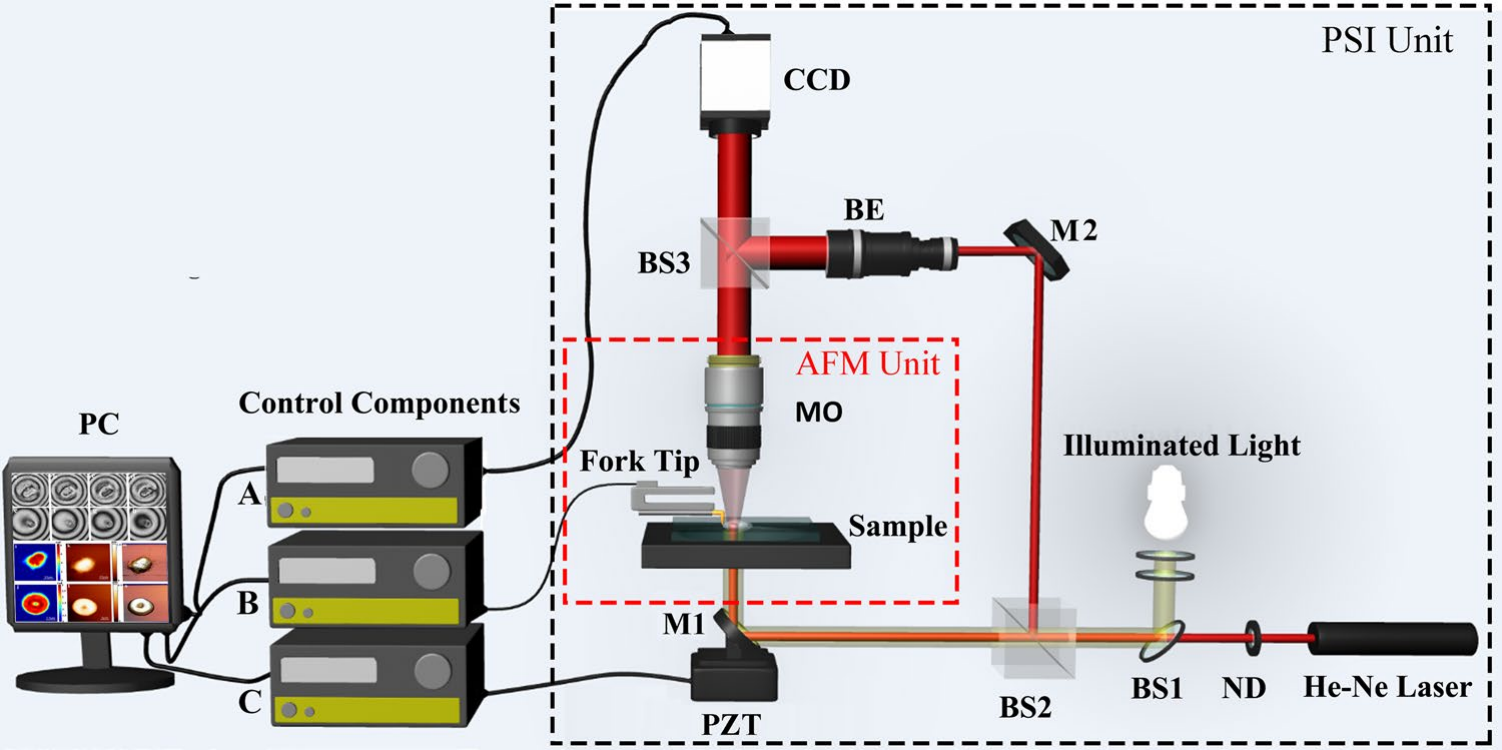
Schematic of PSI/AFM based refractive index measurement system, in which AFM (MultiView 4000, Nanonics, Israel) was equipped with an Olympus BX51 microscope, PSI unit was attached to the microscope. BS1, BS2, BS3: beam splitter; ND: neutral density filter; MO: microscopic objective; BE: beam expander; PZT: piezoelectric ceramics transducer; M1, M2: mirror; Control Components (**A**) Phase-shifting interferogram acquirement; (**B**) AFM scanning control and morphological information acquirement; (**C**) Phase-shifting control.

In this study, we constructed a PSI/AFM based cell refractive index distribution measurement system, in which AFM (MultiView 4000, Nanonics, Israel) was equipped with an Olympus BX51 microscope, and PSI unit was attached to the microscope, as shown in Fig. 1. First, to achieve the quantitative phase distribution of single cell, a Mach–Zehnder interferometer based phase-shifting phase measurement system was chosen. A frequency stabilized He-Ne laser with wavelength of 632.8 nm was utilized as the illumination source, in which the laser beam was split into a transmission beam and a reflection beam through a beam splitter (BS2); the transmission beam was reflected by a mirror (M1) attached to a piezoelectric ceramics transducer (PZT), which was utilized as the phase-shifting inducer, and then modulated by sample and passed through the microscopic objective (MO) as the object beam; the reflection beam was reflected by another mirror (M2), and then expanded by a beam expander (BE) as the reference beam. Second, two beams were overlapped to form interferogram and then captured by a CCD, in which the magnification and numerical aperture (NA) of MO were equal to 25× and 0.40, respectively; the size of CCD was 1024 (V) × 1280 (H) pixels and the pixel size was 5 μm × 5 μm. In addition, the magnification of PSI system 51×, so the corresponding pixel size was equal to 98 nm × 98 nm.

Following, we introduced four-step PSI, a high accuracy phase measurement technique, to perform phase retrieval of single cell^22, 23^, in which four-frame phase-shifting interferograms *I*_1_(*x*, *y*), *I*_2_(*x*, *y*), *I*_3_(*x*, *y*) and *I*_4_(*x*, *y*) with the phase shifts of 0, π/2, π, 3π/2 were respectively captured by a CCD, which was executed by the control system, thus the quantitative phase of measured cell can be expressed as

1 $$\phi (x,y)=\arctan \frac{{I}_{4}(x,y)-{I}_{2}(x,y)}{{I}_{1}(x,y)-{I}_{3}(x,y)}$$

Subsequently, we achieved cellular morphology *I*(*x*, *y*) with AFM imaging system, in which the tip size of probe was 10 nm. For Jurkat cell imaging, the scanning range was set as 50 μm × 50 μm corresponding to 512 × 512 pixels, so the pixel size was equal to 97.6 nm × 97.6 nm. For red blood cell imaging, the scanning range was set as 15 μm × 15 μm corresponding to 512 × 512 pixels, and the pixel size was equal to 29.3 nm × 29.3 nm. Specially, to perform the combination of phase information achieved with PSI system and height information achieved with AFM system, it was needed to perform the size matching of above two images respectively achieved from above two systems in advance. To address this, we first set a location point of measurement area marked with a symbol, and then captured a sequence of four-frame phase-shifting interferograms to achieve the phase information. Second, the tip of AFM system was moved to the location point of measurement area, and then execute the scanning to achieve the morphological information. Third, by performing the interpolation magnification operation, we can achieve the accurate size matching of phase image and AFM image.

### Quantitative refractive index measurement by combining cellular phase distribution and morphology

Typically, for each point of measured sample, the relationship between the phase and height can be expressed as

2 $$h(x,y)=\frac{\phi (x,y)\times \lambda }{2\pi (n(x,y)-{n}_{0})}$$

where *n*(*x*, *y*) and *n*_0_ denoted the cellular refractive index and surrounding medium refractive index, respectively. As described in Eq. (1), we can achieve the quantitative phase distribution with four-step PSI technique; subsequently, the accurate height information of single cell can be measured with AFM imaging. After that, by performing the size matching of two images, the quantitative refractive index distribution of single cell can be calculated from Eq. (2) easily.

### Sample preparation

Peripheral blood samples were achieved from a local hospital (volunteers who attended as altruist donors to the blood bank of the local hospital). Red blood cells (RBCs) were separated from peripheral blood by using RBCs separation fluid as described by Manufacturer’s instructions (Sigma-Aldrich, St. Louis).

Jurkat cells, an immortalized line of human T lymphocyte cells, possessing a relatively simple structure with a large nucleus and small amount of cytoplasm, were purchased from Cell bank (Sun Yat-sen University, China), and then cultured in RPMI-1640 medium containing 10% fetal bovine serum (Hyclone, USA), and 1% of the antibiotics Penicillin G (100 units/mL)/Streptomycin (100 μg/mL) (Gibco, USA) in the cell incubator with the temperature of 37 °C and CO_2_ of 5%.

To facilitate the evaluation during Daunorubicin (DNR)-induced Jurkat cell apoptosis, we defined three stages of cell apoptosis based on the drugs treatment time (i) in the early stage of cell apoptosis, drugs treatment time was less than 24 h; (ii) in the middle stage of cell apoptosis, drug treatment time was between 24 h and 48 h; (iii) in the late stage of cell apoptosis, drug treatment time was more than 72 h. For comparison, three group samples were prepared as following: (i) Jurkat cells were cultured with 2.0 umol/L DNR (Pharmacia Italia SPA, Italy) for 24 h as experimental group 1; (ii) Jurkat cells were cultured with 2.0 umol/L DNR for 48 h as experimental group 2; (iii) Jurkat cells were cultured with 2.0 umol/L DNR for 72 h as experimental group 3; (iv) Jurkat cells were cultured without any treatment as control group.

For optical phase measurement and AFM imaging study, double-distilled water suspensions were spread onto glass cover slides that were pre-treated with poly-L-lysine (Sigma-Aldrich, St. Louis) and air-dried at room temperature.

All the animal use protocols and the experiments in the method section were reviewed and approved by the Experiment Committee of South China Normal University and Use Committee of Sun Yat-sen University. And the consent was obtained from all subjects.

### Raman spectroscopy

To determine the quantitative biochemical component changes during DNR-induced Jurkat cell apoptosis, we employed a Raman spectrometer (InVia + Plus, Renishaw, UK) attached to a Leica upright microscope equipped with a 50× objective and a 632.8 nm laser with the power of 50 mW to collect Raman spectra of individual cell, in which ten different areas were randomly measured within a cell and the integration time for acquiring a Raman spectrum was 10 sec in all experiments. The spectral resolution was set as 0.99 cm^−1^. Each Raman spectrum was achieved from the average of ten individual cells in the scanning range of 600–1800 cm^−1^, which was described as the average Raman spectra in this study. In addition, the procedure of Raman data processing was as following: (1) Fluorescence background of Raman spectrum was estimated by using the fifth order polynomial fitting and subtracted from the original spectrum; (2) Raman spectrum was smoothed by using Savitzky–Golay method and performed the normalization processing, and all above data processing was performed with the software of MATLAB and Origin.

### Fluorescence imaging

Fluorescence imaging was utilized to determine the location of cellular nucleus during DNR-induced Jurkat cell apoptosis, in which Hoechst 33258, a blue nucleic acids fluorescent dye, was chosen to label cellular nucleus, following imaged with a fluorescence microscope (Nikon Ti-U, Japan) at 350 nm excitation light and a 40× objective (NA = 0.13, Nikon), as well as an available additional 1.5× magnification.

## Results and Discussion

### The accuracy of PSI/AFM based refractive index measurement system

**Figure 2:**
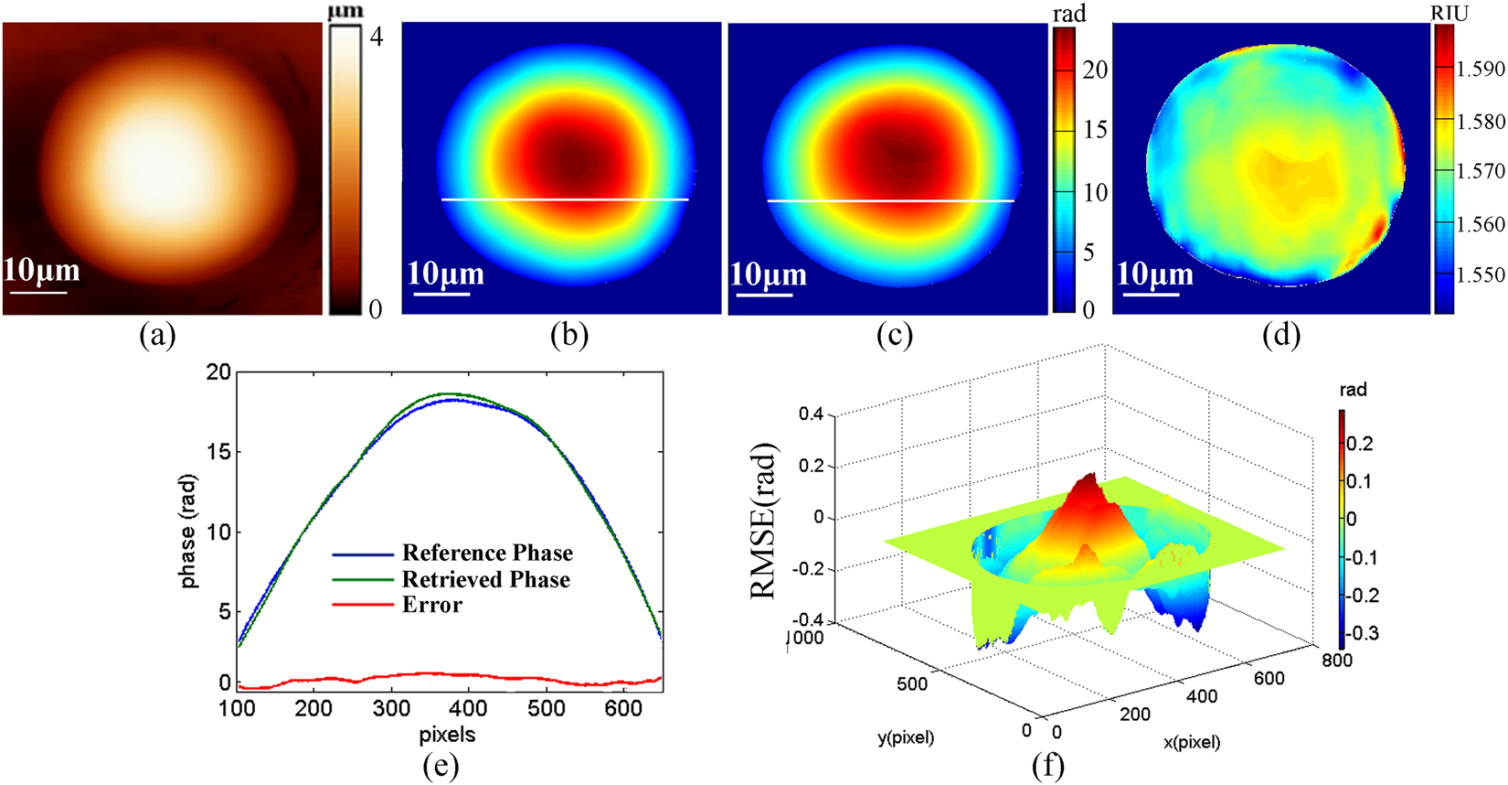
Experimental results of a homogeneous hemispherical particle on the light guide plate achieved with the proposed PSI/AFM based refractive index measurement system, in which the height distribution (**a**) and the retrieved phase (**b**) were respectively achieved by AFM imaging and PSI technique; the reference phase (**c**) achieved from Eq. (2) with the nominal refractive index and height distribution; the refractive index (RI) distribution (**d**) directly calculated from Eq. (2); (**e**) the phase distribution curves of the white line marked in (**b)** and (**c**), as well as and the corresponding error between the reference phase and the retrieved phase; (**f**) the error of phase retrieval achieved with PSI system.

In this section, we first performed the accuracy estimation of the proposed PSI/AFM based refractive index measurement system. Due to the proposed refractive index measurement system was consisted of PSI system with microscale resolution and AFM imaging system with nanoscale resolution, that is to say, the measurement error induced by AFM system could be ignored compared with PSI system. Therefore, the accuracy of refractive index measurement was mainly decided by the accuracy of PSI system. To perform the accuracy estimation of PSI system, we first employed a homogeneous hemispherical particle with nominal refractive index of 1.567 RIU on the light guide plate as the sample. Using the proposed PSI/AFM system, we achieved the height distribution and phase of measured sample, as shown in Fig. 2(a) and (b), respectively. Subsequently, the quantitative refractive index (RI) distribution of the hemispherical particle can be calculated from Eq. (2), as shown in Fig. 2(d). For comparison, by utilizing the nominal refractive index and the achieved height information, we can achieve the reference phase (REF) of the hemispherical particle from Eq. (2), as shown in Fig. 2(c). In addition, Fig. 2(e) and (f) presented the error of phase retrieval. It was found that the root mean square error (RMSE) of the difference between the REF and the retrieved phase and peak to valley error (PVE) were respectively 0.0997 rad and 0.6304 rad, and the corresponding RMSE and PVE of refractive index measurement were 0.0025 RIU and 0.0625 RIU, respectively. From these results, we can conclude that the proposed PSI/AFM system should be a useful tool for quantitative refractive index distribution measurement.

### Phase maps and morphologies of single Jurkat cell and red blood cell

**Figure 3:**
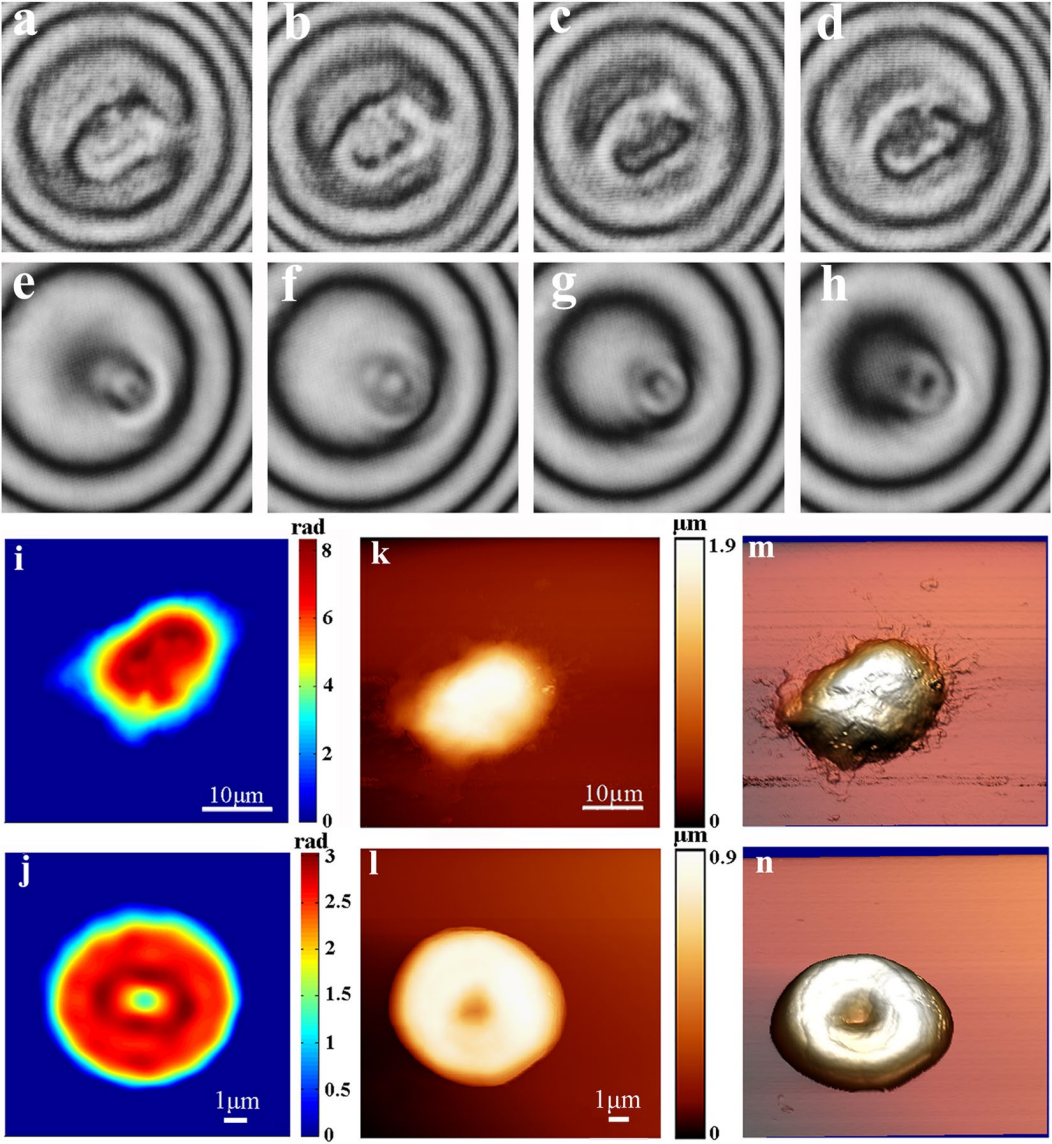
Four-frame phase-shifting Interferograms of Jurkat cell (**a–d**) and RBC (**e–h**) respectively with the phase shifts (**a,e**) 0; (**b,f**) π/2; (**c,g**) π; (**d,h**) 3π/2. Phase maps of Jurkat cell (**i**); RBC (**j**); Height distributions of Jurkat cell (**k**) and RBC (**l**); 3D morphologies of Jurkat cell (**m**) and RBC (**n**).

Using the above PSI system, we can achieve accurate phase distribution of single cell. Fig. 3(a)–(d) and (e)–(h) respectively presented four-frame phase-shifting interferograms of single Jurkat cell and red blood cell with the spatial phase shifts of 0, π/2, π, and 3π/2; Fig. 3(i) and (j) showed the phase distributions of single Jurkat cell and red blood cell, respectively. Moreover, using AFM imaging method, we achieved height distributions of single Jurkat cell and red blood cell, as shown in Fig. 3(k) and (l), respectively. For direct visualization, we also presented the corresponding 3D topographies of single Jurkat cell and red blood cell, as shown in Fig. 3(m) and (n), respectively.

Next, to achieve the accurate refractive index distribution of single cell, it was needed to perform the fusion of phase map and morphology information. That is to say, the spatial matching of phase map and morphology information will be a critical step. To address this, we first calibrated the constructed PSI/AFM system by the linear interpolation algorithm. Subsequently, the quantitative refractive index distribution of single cell can be calculated from Eqs (1) and (2). As we know, the accuracy of refractive index measurement depended on the accuracy of both phase retrieval and height measurement. In our PSI system, the sampling interval was calibrated to 98 nm × 98 nm, which was significantly larger than AFM imaging system. Therefore, the accuracy of refractive index measurement was mainly decided by the accuracy of phase retrieval. In addition, to improve the reliability, the phase maps of each cell type (Jurkat cell and red blood cell) were measured by an average of 5 times.

### Refractive index distributions of single Jurkat cell

**Figure 4:**
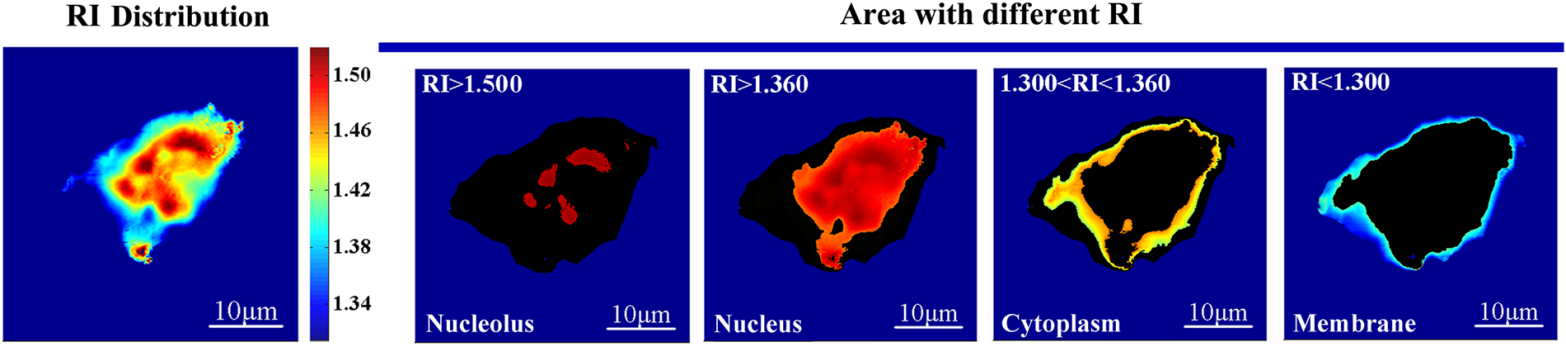
Refractive index distribution of single Jurkat cell, in which the refractive index distribution was coded with pseudocolor, the areas of RI > 1.360 were marked as nucleus, specially, RI > 1.500 corresponding to nucleolus; the areas of 1.360 > RI > 1.300 and RI < 1.300 were respectively marked as cytoplasm and membrane.

**Figure 5:**
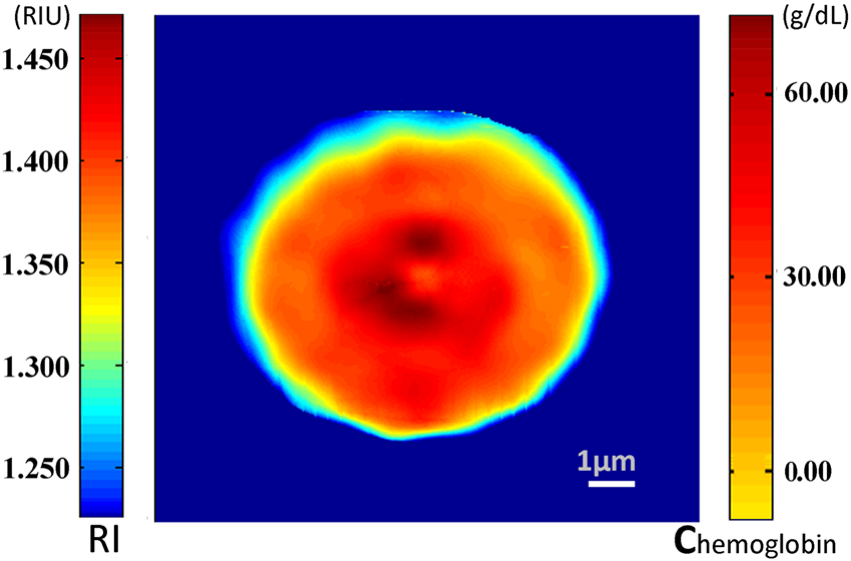
Refractive index and the corresponding hemoglobin concentration distribution of single red blood cell.

Based on the constructed PSI/AFM system, and then performing the data fusion of phase distribution and morphology information, we can conveniently achieve the refractive index distribution of single cell, as shown in Fig. 4 and Fig. 5. Clearly, there was obvious difference of refractive index distribution between Jurkat cell (Fig. 4) and red blood cell (Fig. 5).

As we knows, Jurkat cell reveals a relatively simple structure, in which most intracellular areas are occupies by nucleus. Typically, the average refractive index of cellular nucleus was in the range of 1.360 to 1.391 RIU^1, 22–25^. In response, from Fig. 4, we can see that refractive index of most intracellular middle areas were larger than 1.360 RIU, further indicating that most middle areas of Jurkat cell were occupied by nucleus. Specially, it was observed that the refractive index of a few areas were larger than 1.500 RIU, reflecting the location of high density biochemical components in nucleolus, such as deoxyribonucleic acid (DNA), ribonucleic acid (RNA) and ribonucleoprotein due to it was reported that the average refractive index of DNA and ribonucleoprotein or RNA in nucleolus were in the range of 1.530 to 1.560 RIU^26–28^ and 1.540 RIU^4^. Based on these results, we can conclude that the areas that refractive index was greater than 1.500 RIU should be the distribution areas of nucleolus. In addition, previous research showed there were various organelles and biochemical components in cytoplasm, such as mitochondria (RI ≈ 1.400–1.420 RIU^24, 29^, ribosome (RI ≈ 1.330–1.340 RIU)^30^, glucose (RI = 1.340 RIU)^31^ *et al*. If the refractive index of these organelles or biochemical components in cytoplasm can be employed as the corresponding marker, we can achieve the identification of cellular organelle or biochemical components with the proposed PSI/AFM system. Typically, the average refractive index of cytoplasm was in the range of 1.360 to 1.380 RIU^4, 24, 32^, so the areas that refractive index was lower than 1.360 RIU should be identified as the non-nucleus area. Further, we can see that in cellular edge, the refractive indexes were lower than 1.300 RIU, one possible explanation that the major biochemical components in cellular edge were cellular membrane due to its refractive index was in the range of 1.080 to 1.160 RIU^33^.

Following, from the above refractive index distributions of nucleus and cytoplasm, we can calculate the volume of cellular nucleus. As we know, the nucleus and cytoplasm are the major cellular components thus we have that

3 $${h}_{1}(x,y)+{h}_{2}(x,y)={h}_{0}(x,y)$$

4 $${n}_{1}\times {h}_{1}(x,y)+{n}_{2}\times {h}_{2}(x,y)=n(x,y)\times h(x,y)$$

where *h*_1_(*x*, *y*) *h*_1_(*x*, *y*) and *h*_2_(*x*, *y*) respectively denoted the height of nucleus and cytoplasm, *h*_0_(*x*, *y*) represented the total cellular height, *n*(*x*, *y*) represented the cellular refractive index. If n_1_ ≈ 1.361–1.391 RIU denoted the average refractive index of nucleus, n_2_ ≈ 1.360 RIU was the average refractive index of cytoplasm, so the areas that refractive indexes were greater than 1.391 RIU can be thought as the location of nucleus. According to the following equation

5 $${V}_{n}={R}^{2}\times \sum _{x,y}\,{h}_{n}(x,y)\,,(n=0,1,2)$$

where *Vn*(*n* = 0, 1, 2) denoted the volumes of nucleus, cytoplasm and whole cell, *h*_*n*_(*x*, *y*) were the corresponding heights, R was the corresponding sampling interval was 97.6 nm. Regardless of the influence of other organelles, it was found that the volume of Jurkat cell was equal to 393.80 fL (μm^3^); and the volume of nucleus was 305.31 ± 9.01 fL, which accounted for about 77.53 ± 2.29% of total cellular volume.

### Refractive index and hemoglobin concentration distribution of single red blood cell

As we know, RBCs, being the absence of nucleus and organelles, was filled with hemoglobin at a constant concentration throughout. Previous research has showed that there was a significant difference of refractive index distribution between the healthy and infected RBCs, in which the healthy RBCs had relatively homogeneous distribution in refractive index while the infected RBCs revealed non-homogeneous distribution in refractive index throughout cytoplasm, so both the refractive index and morphology information of RBCs can be employed as the characteristic parameters for disease diagnosis such as malaria and anemia^5, 10^.

Typically, the average refractive index of red blood cell was in the range of 1.370–1.420 RIU^5, 10, 12, 34^. From Fig. 5, we can see the refractive index distribution of RBCs was greatly different from Jurkat cell. In most areas of RBCs, refractive indexes were in the range of 1.370–1.420 RIU while the refractive index of the concave areas were larger than 1.420 RIU. This was because some biochemical components with high density, such as lipid (RI ≈ 1.450–1.480 RIU)^32, 33^, chromosome and residual nucleus compositions, usually appeared around the concave area of RBCs. However, in the edge areas of RBCs, the refractive indexes were less than 1.370 RIU, one probable explanation was that the concentration of water around cellular membrane was increased.

Like the above Jurkat cell, we also can achieve the volume of RBCs from Eq. (5). The calculation result showed that the volume of single RBCs was equal to 24.52 ± 1.43 fL. Thus, by using the equation $\,nRBC={n}_{0}+\alpha {C}_{hemoglobin}$, where *n*_0_ = 1.335 was the refractive index of cell fluid without hemoglobin and *α* = 0.00193*dLg* ^−1^, we can determine the concentration distribution of hemoglobin, as shown in Fig. 5. It can be seen that the distribution of hemoglobin in RBCs was very homogeneous, indicating our result was consisted with previous research.

### Refractive index distribution changes during Jurkat cell apoptosis

**Figure 6:**
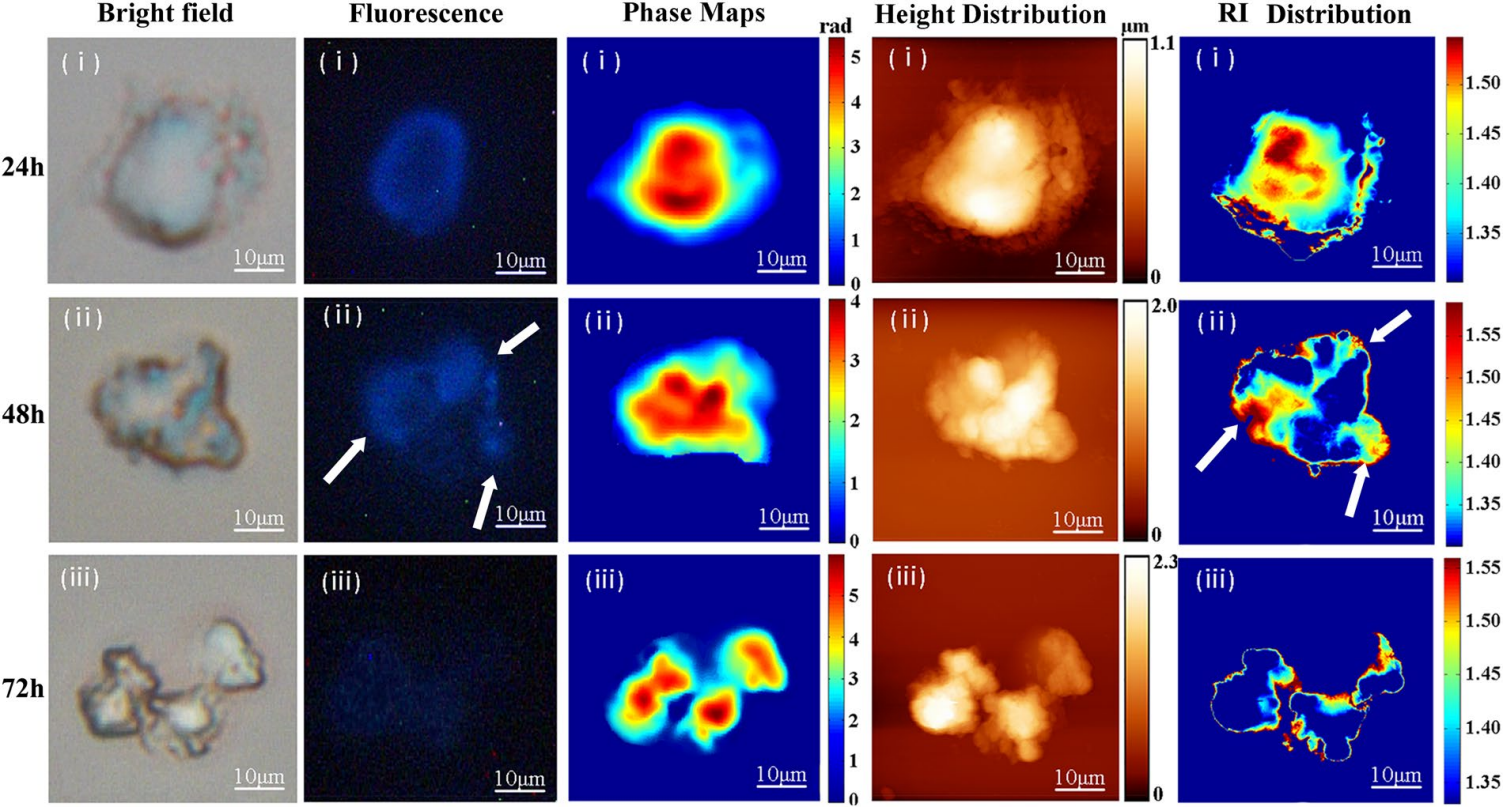
Experimental results of Jurkat cells treated with DNR in the (**i**) early stage; (**ii**) middle stage; (**iii**) late stage, in which the first column denoted the bright images of Jurkat cells, the second column were the fluorescence images of cellular nucleus stained with Hoechst 33258, the third column represented the cellular phase maps, the fourth column denoted the cellular height distribution, and the fifth column were the cellular refractive index distributions.

In this section, the experimental results of Jurkat cells treated with DNR in the early stage, middle stage and late stage of cell apoptosis were respectively shown in Fig. 6(i)–(iii). Note that in the early stage of cell apoptosis (the first row), only a few areas that RIs > 1.360 RIU corresponding to nucleus component can be seen in the cellular edge with wrinkle fluctuation; in the middle stage (the second row), along with the morphologic collapse, the nucleus component was spread to the cellular edge; in the late stage, Jurkat cell was broken in morphology, and several apoptotic bodies appeared, and the nucleus were observed only in the edge of apoptotic bodies. Clearly, whether from the morphology, phase map or bright field image, it was impossible to achieve the change of intracellular biochemical components. Previous research has demonstrated that along with the increasing of drugs treatment time, the change of nucleus condensation and fragmentation during DNR-induced Jurkat cell apoptosis can be observed by fluorescence imaging method^35, 36^. Importantly, using our PSI/AFM system, the quantitative change of refractive index distribution during Jurkat cell apoptosis can be directly visualized, as shown in Fig. 6(ii), in which the areas marked with white arrow presented the nucleus condensation and fragmentation. From these results, we can conclude that PSI/AFM system might become a useful tool for revealing cell structure and pathological state.

**Figure 7:**
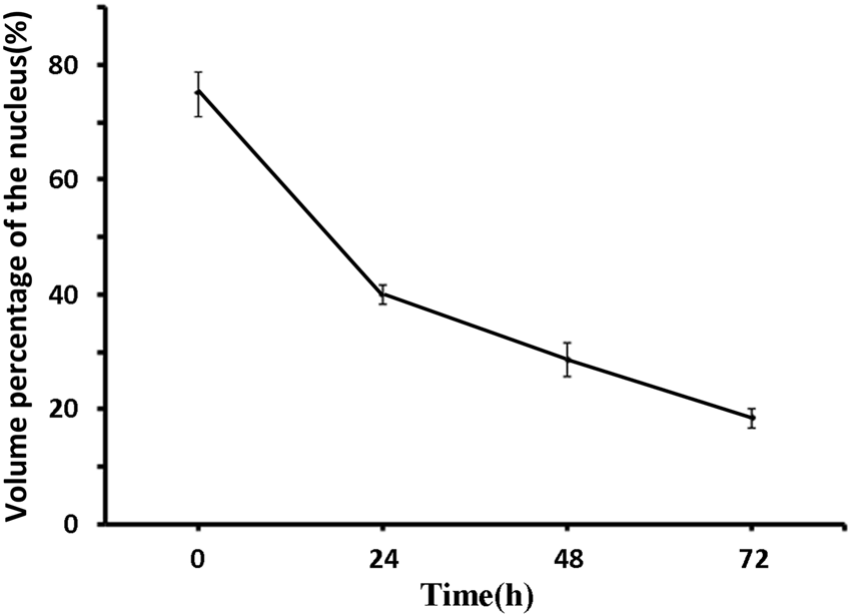
The change of volume percentage of nucleus during DNR-induced Jurkat cells apoptosis achieved by the proposed method.

Further, we can calculate the volume percentage of nucleus during DNR-induced Jurkat cells apoptosis, as shown in Fig. 7. It was found that the volume percentage of nucleus during DNR-induced Jurkat cell apoptosis were 77.53 ± 4.29% (without any treatment), 39.12 ± 2.74% (early stage), 27.2 ± 1.36% (middle stage) and 17.59 ± 1.82% (late stage), respectively.

**Figure 8:**
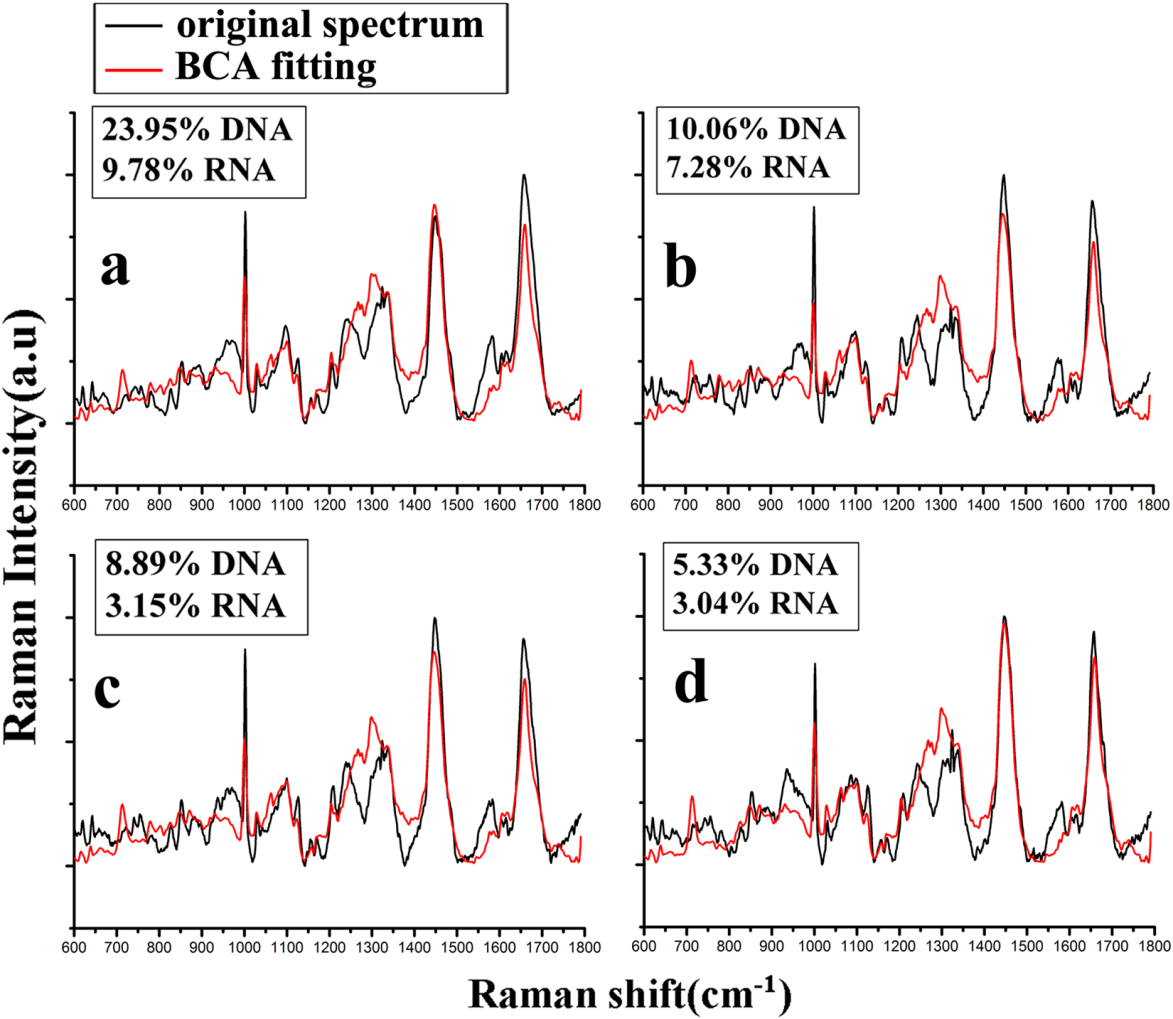
The relative contents of DNA and RNA of Jurkat cells achieved by BCA fitting of Raman spectra, in which Jurkat cells were treated with DNR-induced apoptosis (**a**) without treatment; (**b**)in the early stage; (**c**) in middle stage; (**d**) in late stage.

To verify the validity of refractive index measurement with the proposed method, we also gave the content changes of DNA and RNA during DNR-induced Jurkat cells apoptosis through the biochemical component analysis (BCA) of Raman spectrum of Jurkat cells. As described in previous reports^37, 38^, seven basic components, actin, albumin, triolein, phosphatidylcholine, DNA, RNA, and glycogen, were employed as the main biochemical components of biological cells, in which actin and albumin were attributed to proteins, triolein and phosphatidylcholine were attributed to lipids, glycogen was attributed to polysaccharides, and DNA and RNA were attributed to nucleic acids. By performing the BCA fitting of Raman spectrum, the relative content of cellular biochemical components can be achieved. Accordingly, Fig. 8 showed the relative contents of DNA and RNA during Jurkat cells apoptosis by the BCA fitting of Raman spectrum. We can see that the contents of DNA and RNA were reduced with the increasing of DNR treatment time.

**Figure 9:**
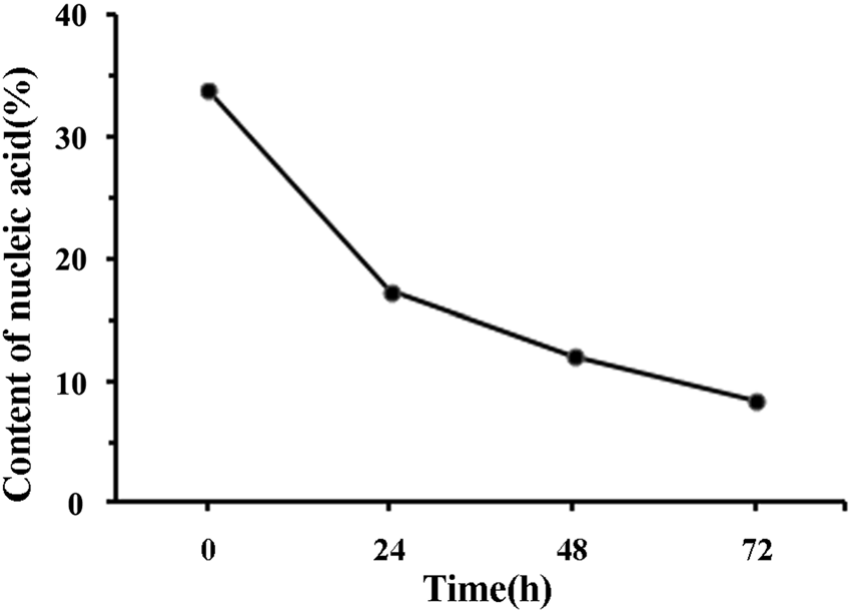
The content change of total nucleic acid of Jurkat cells with DNR treatment time.

Further, we also calculated the content change of total nucleic acid (DNA + RNA) with DNR treatment time, as shown as in Fig. 9. Like the volume percentage, it was observed that the content of nucleic acid was also reduced with the increasing of DNR treatment time. In the early stage, the volume percentage was reduced from 77.53 ± 4.29% to 39.12 ± 2.74%; accordingly, the content of total nucleic acid was reduced from 33.73 ± 3.59% to 17.34 ± 2.12%. In the middle stage, the volume percentage was reduced from 39.12 ± 2.74% to 27.20 ± 1.36%, accordingly, the content of total nucleic acid was reduced from 17.34 ± 2.12% to 12.04 ± 1.68%. In the last stage, the volume percentage was reduced from 27.2 ± 1.36% to 17.59 ± 1.82%; in response, the content of total nucleic acid was reduced from 12.04 ± 1.68% to 8.37 ± 1.25%. Importantly, these results showed that the change of cellular biochemical component achieved with our PSI/AFM based refractive index measurement system was coincident with the BCA fitting data of Raman spectrum, and further indicating the proposed method was suitable for elucidating cellular composition and structure.

## Conclusion

In this study, we constructed a novel PSI/AFM based cell refractive index distribution measurement system, in which the accurate phase map of single cell was retrieved with PSI, and the cellular morphology with nanoscale resolution was achieved with AFM imaging. Based on the proposed PSI/AFM system, we achieved the quantitative refractive index distributions of Jurkat cell and red blood cell, respectively. Different with current refractive index measurement methods (quantitative phase imaging, TPM and TIE), in which cell morphology was approximated as the spherical object or the accuracy of phase measurement depended on the information amount of scattering field collection, the proposed PSI/AFM method can retrieve both the accurate cell phase and height information, so the quantitative refractive index distribution of single cell can be presented. Moreover, the volume of cellular nucleus can be calculated, and then the volume percentage of cellular nucleus in the whole cell can be achieved. Importantly, the change of refractive index distribution during DNR-induced Jurkat cell apoptosis, reflecting the content change of intracellular biochemical components, was directly visualized, and then the quantitative volume change of cellular nucleus during DNR-induced Jurkat cell apoptosis was also achieved. To our knowledge, there is no report on studying the quantitative cellular volume change during apoptosis. Now, this novel PSI/AFM measurement system will allow us to directly visualize the cellular refractive index distribution and volume change during apoptosis. More importantly, the above results were consistent with Raman spectral analysis, which can achieve only the relative content of intracellular biochemical components but lacking of cellular quantitative volume information, further indicating the proposed PSI/AFM system was suitable for elucidating cellular composition and structure, and this will facilitate its application in cytobiology or disease diagnosis.
